# 

**Published:** 1824-03-01

**Authors:** 


2. The Circulation of the Blood - - - - - - - - - - - 966

Bibliographical Record - - - - - - - - - - - - - - 1015

XIII.

Miscellaneous Intelligence, &c. &c. - - - - - - - - - 1019

List of Additional Subscribers - - - - - - - - - - - 1022

---

### TO THE PUBLIC.

Almost the whole of the back Numbers of this Series being now out of print, it has been determined on to commence a New Series with the next Number, *but without any deviation from the plan of the present Series.* This will enable New Subscribers to commence with complete sets, without any expence of purchasing back Numbers; while to the holders of the present Series, a spare Title Page will be given with each Volume, so as to form an unbroken Series with the present.

Each Number of the New Series will contain 250 pages, instead of 224, without any increase of price, and all *Extra-Limites* papers shall be, as hitherto, beyond the above boundary, and gratis to the Public. Plates and illustrative wood cuts shall accompany each Number, and in short, no labour or expence will be spared to repay the unexampled encouragement which the public has extended to this Review.

*London,*
*25th February,* 1824.

### XI.

### BIBLIOGRAPHICAL RECORD;

OR

### *Works received for Review since last Quarter.*

1. An Essay on the Absorbent Vessels; shewing that their action is not liable to be influenced by the Artificial Agents commonly applied. By Henry Searle, Surgeon, London. Octavo, sewed, pp. 26. London, 1823. Price 2s. 6d.

---

2. The Philadelphia Journal of the Medical and Physical Sciences, No 24, in exchange.

---

3. A Probationary Essay on the Use and Effects of Mercury in the Treatment of Syphilis; submitted by Authority of the President and Council, to the Examination of the Royal College of Surgeons of Edinburgh, when Candidate for Admission into their Body. By David Johnston, M.D. Extraordinary Member of the Royal Medical Society. October 1823.

☞ *Very creditable to Dr. Johnston's erudition and judgment.*

---

4. Letters on the Prevention and Cure of Diseases peculiar to Hot Climates, intended for the Use of Commanders of Ships, and Persons about to settle in, or visit such Climates; with Remarks on the Use of the Tourniquet, &c. &c. By James Boyle, Esq. Surgeon, &c. Author of a Treatise on the Epidemic Cholera of India. 2s. 6d.

☞ *This little Work contains plain and concise directions for the Class of people abovementioned; and to them will prove useful, in the absence of medical advice.*

---

☞ The following Work came too late for our Bibliographical Record of last quarter, and was only entered in the advertising sheet.

5. An Engraved Representation of the Anatomy of the Human Ear, exhibiting, in one view, the External and Internal Parts of that Organ, *in situ*, accompanied with a Plate of Outlines and References, with copious Explanations. To which are added, Surgical Remarks on introducing the Probe and Catheter into the Eustachian Tube by the Nostril—on puncturing the Membrana Tympani, &c. &c. The whole designed as a Guide to Acoustic Surgery. By Thomas Buchanan, C. M. Surgeon to the Hull Dispensary for Diseases of the Eye and Ear. Folio, pp. 40, two plates. London, 1823. Price 12s. 6d.

☞ *This valuable Work will be reviewed in our next number.*

---

6. Observations on the State of the Peasantry of the West of Ireland, during the Epidemic Fever, &c. in the months of July, August, and September, 1822. Dedicated to His Excellency the Lord Lieutenant. By De Burgh Birch, M.D. Licentiate of the Royal College of Surgeons in Ireland. *Second Edition.* Octavo, sewed, pp. 24. London, 1823. Price 2s.

---

7. On the Nature and Treatment of the Distortions to which the Spine and the Bones of the Chest are subject. With an Enquiry into the Merits of the several Modes of Practice which have hitherto been followed in the Treatment of Distortions. Illustrated by plates in folio. Octavo, pp. 293. By John Shaw, Surgeon, and Lecturer on Anatomy. London, Dec. 1823. Longman and Co. 10s. 6d.

8. Tears for Pity. By WILLIAM BARRET MARSHALL. Octavo, pp. 162. London, Dec. 1823.

☞ *We are not competent, nor is it our place, to say any thing of the poetry; but the preface* evinces a mind well stored with useful knowledge, and highly imbued with those principles which, in all ages, have done honour to human nature. When the subject of this preface comes before us, we shall take an opportunity of making some extracts for the gratification of our readers.*

* "Containing an enquiry into some of the prejudices against the medical profession; more particularly that which alledges infidelity as the natural tendency of physiological pursuits."

---

9. Observations on Prison Discipline, exemplified by the Tread-Mill and Dietary adopted in the Nottinghamshire House of Correction at Southwell. By BENJAMIN HUTCHINSON, Surgeon to the Establishment, &c. Octavo, sewed, pp. 55. Newark, Dec. 1823. 2s. 6d.

---

10. Cases of Inflammation of the Vascular and Cellular Structures. By ANDREW DUNCAN, Jun. M.D. 8vo.

☞ *Not sold separately.*

---

11. Transactions of the Medico-Chirurgical Society of Edinburgh. Instituted August 2d, 1821. Octavo, pp. 697, four coloured plates. Price 18s. 6d. Edinburgh. 1823.

☞ *This interesting stranger from the North shall have due introduction, and, we fear not, hospitable reception in the South. We reserve the review of it for our next number, the first of a New Series.*

---

12. The Principles of Forensic Medicine, systematically arranged, and applied to British Practice. By JOHN GORDON SMITH, M.D. Lecturer on Political Medicine. *Second Edition,* greatly enlarged. 8vo, pp. 579. Price 16s 6d. Underwoods, 1824.

---

13. On the comparative Virtues of the different Kinds of Sarsaparilla; to which is added, an Appendix on Cinchonine, Quinine, and other new Vegetable Principles. By Mr. JOHN POPE, Operative Chemist, of Oxford Street. Octavo, pp. 21.

☞ *This will be noticed in another part of the Journal, when reviewing Magendie's Formulæ.*

---

14. Remarks on the Dangers and Duties of Sepulture; or, Security for the Living, with Respect and Repose for the Dead. By a Fellow of the Massachusett's Medical Society. Octavo, sewed, pp. 74. Boston, (America,) 1823.

☞ *Our author's motto is taken from Seneca—"Non defunctorum causa, sed vivorum inventa est sepultura." In accordance with this principle, he strenuously reprobates the practice now creeping into use beyond the Atlantic, of burying the dead in churches. He considers this mode of sepulture as highly dangerous to the health of the living, and draws arguments and examples from all ages, with considerable erudition and force of persuasion.*

---

15. An Essay on an improved Method of Cutting for Urinary Calculi; or, the Posterior Operation of Lithotomy. By W. W. SLEIGH, Member of the Royal College of Surgeons, and Lecturer on Anatomy and Surgery in London. 8vo, pp. 106, two plates. Anderson, London. Price 6s. bds. 1824.

16. Essays on Various Subjects connected with Midwifery. By W. P. Dewees, M. D. &c. Octavo, pp. 480. Philadelphia, 1823.

☞ *We shall take as early notice as possible of Dr. Dewees' Essays. In the mean time, we have to inform the Profession that a few copies of this Work are to be had of Mr. Millar, 69, Fleet Street, London.*

---

17. Essays on Fevers and other Medical Subjects. By Thomas Miner, M.D. and William Tulley, M D. 8vo. pp. 484. Middle-town, (Connecticut, America,) 1823.

---

18. Biographical Sketch of the late Matthew Baillie, M.D. By James Wardrop, Esq. Surgeon Extraordinary to the King. Octavo, pp. 17. 1824.

---

19. An Exposition of the Dangers of Interment in Cities; illustrated by an Account of the Funeral Rites and Customs of the Hebrews, Greeks, Romans, and Primitive Christians, &c. compiled chiefly from the Works of Vicq. D'Azyr and Professor Piattoli, with Additions. By Felix Pascalis, M. D. &c. Octavo, pp. 167. New York, 1823.

---

20. Transactions of the Medical Society of the State of New York, for the Year 1823. With an Appendix, containing a System of Medical Ethics. Octavo. New York, 1823.

☞ *The System of Medical Ethics is drawn up by that zealous and able medical philosopher, Dr. Felix Pascalis. Would to Heaven that his brethren, on both sides of the Atlantic, would adhere to the rules which he has laid down, and which are creditable to the head and heart of their author. We shall take an early opportunity of noticing this work.*

---

21. Address, delivered before the New York County Medical Society, on the 11th of November, 1822. By Felix Pascalis, M.D. Censor of the State Medical Society of New York, &c. &c. Octavo, 1823.

☞ *This discourse is on the nature of the late epidemic fever. Dr. P. does not consider it as contagious, but depending on a vitiated condition of the atmosphere, produced by exhalations or deleterious gases. These, he thinks, interrupt the functions of the lungs primarily, and cause pestilential diseases.*

---

22. Practical Observations on the Removal of every Species and Variety of Cataract by Hyalonyxis, or Vitreous Operation, illustrated by Cases; with critical and general Remarks on the other Methods employed. By John Bowen, M. D. Fellow of the Royal College of Physicians of Edinburgh, Member of the Royal College of Surgeons of London, &c. &c. Octavo, pp. 120. Price 6s. Callow and Wilson, London, Jan. 1824

---

23. An Essay on Apparitions, in which their appearance is accounted for by causes wholly independent of preternatural agency. By John Alderson, M.D. Senior Physician to the Hull General Infirmary, &c. 8vo. pp. 53. New Edition, revised and corrected.

---

24. Dissertatio Inauguralis Medica Sistens Experimenta Nonnulla Circa Vitam Arteriarum et Circulationem Sanguinis per vasa Collateralia. Auctore Frederico Guilielmo Oppenheim, M. D. Hamburgensi. 4to. with a plate. Manheim, 1823.

☞ *Thanks to our ingenious young friend, whose thesis does honour to himself, and the celebrated professors of his Alma Mater, Heidelberg.*

25. Pathological Observations on the Rotated or Contorted Spine, commonly called Lateral Curvature, deduced from Practice. In which are shewn the Causes that produce it; the Reason of its being mistaken for an Incurvation of the Spinal Column; and the Means best adapted to its Prevention and Cure, agreeably to the Principles laid down, and the Author's Experience. By ANDREW DODS, M.D. late of Edinburgh, and Surgeon in the Royal Navy. 8vo. pp. 239, with wood cuts. Cadell, London, 1824. *Dedicated by permission to Sir Astley Cooper, Bart.*

☞ *This is a valuable addition to our knowledge of Spinal Diseases—contains new views of the subject, both as to pathology and treatment—does great credit to the author—and will be useful to the public. We shall give a review of the work in our next number.*

---

26. Pathological Observations. Part I. On Dropsy, Purpura, and the Influenza of the latter end of the year 1822, and beginning of the year 1823; and particularly on the Morbid Changes of the Blood, and their Influence on the Production and Course of these Diseases, illustrated by select Cases and Dissections. By WILLIAM STOKER, M.D. *Ed.* &c. Senior Physician to the Fever Hospital, and House of Recovery in Cork Street, Dublin. 8vo. pp. 244. Dublin, 1824. Price 8s. 8d. boards.

---

27. Observations on Strictures of the Rectum and Colon, and other Affections which diminish the Capacity of the Intestinal Canal in those parts; including Spasmodic Constriction of the Sphincter Ani, Hæmorrhoidal Tumours, Excrescences, and Prolapsus Ani, accompanied with the Mode of Treatment, several Cases, and Engravings. By W. WHITE, Member of the Royal College of Surgeons, and one of the Surgeons to the City Infirmary and Dispensary, Bath. 8vo 4th Edition, much enlarged, pp. 217, plates, price 7s. 6d. London, 1824.

☞ *This Edition appears to be much improved as well as enlarged. We gave a full Analysis of the Work in a former number of this Journal.*

---

28. Dissertatio Medica Inauguralis de Inflammatione Communi; AUCTORE JOANNE ELLIOTSON, M.D. Edinburgi, 1810.

☞ *A highly classical thesis.*

---

29. Practical Observations on the Symptoms, Discrimination, and Treatment of some of the most Important Diseases of the Lower Intestines, and Anus:—particularly including Stricture, Ulceration, Intussusception, and Tumour within the Cavity of the Rectum, &c. &c. &c. illustrated by numerous cases. By JOHN HOWSHIP, Member of the Royal College of Surgeons, &c. &c. &c. 8vo. pp. 282, 3d Edition, with numerous additions. 1824.

☞ *This edition is very considerably enlarged and improved.*

---

30. The Origin of Various Establishments for Conducting Chemical Processes and other Medicinal Preparations at Apothecaries Hall. 8vo. sewed, pp. 21, with a plate. Apothecaries Hall, 1824.

☞ *In this pamphlet, there are some observations (several of them we fear too true) on the adulteration of medicines by private chemists and druggists. They admit, of course, that there are many respectable individuals of this class, in London, "from whom genuine drugs may be purchased."*

---

31. An Essay on the Effects of Iodine on the Human Constitution; with Practical Observations on its Use in the Cure of Bronchocele, Scrofula, and the Tuberculous Diseases of the Chest and Abdomen. By W. GAIRDNER, M.D. 8vo. pp. 64, Underwoods, 1824. Price 3s. 6d. boards.

Adamson, Mr. Surgeon, Rye.
Albert, Dr. Tenby, South Wales.
Baker, James, Esq. Member of the Royal College of Surgeons, Staines.
Berry, Mr. Surgeon, Liphook, Hants.
Blake, Andrew, Esq. Surgeon, Fifth Regiment, Foot.
Bœtticher, Mr. Charles, Surgeon, Ryde, Isle of Wight.
Bucknill, Mr. Samuel, Surgeon, Rugby, Warwickshire.
Catlet, Mr. William, Jun. Grantham, Lincolnshire.
Chapman, Mr. Member of the Royal College of Surgeons, Kirbymoorside.
Curtis, Mr. W. Medical Student, St. Thomas's and Guy's Hospitals.
Crowfoot, W. J. Esq. Halesworth.
Florance, Mr. W. Surgeon, Chichester.
Fortune, Mr. John, Surgeon, Carriacow.
Fraser, W. W. Esq. Deputy Inspector of Hospitals, Gibraltar.
Gozna, Mr. Thomas, Jun. Grantham, Lancashire.
Harrol, Mr. E. Surgeon of Cheshunt.
Hawkins, J. Esq. Surgeon, Laugharne, Caermarthenshire.
Hickman, Mr. C. Assistant Surgeon 2d Batt. Royal Native Infantry.
Hicks, Mr. Surgeon, Conduit-st.
Holden, Mr. J. F.
Holgate, John Wyndham, Esq. Hendon, Middlesex.
Jones, Mr. Jones, Surgeon, Conway.
Jones, Dr. Walter, Fellow of the Royal Medical Society of Edinburgh; Haverford West, Pembrokeshire.
Kingsley, Mr. William, Licentiate of the Royal College of Physicians of Ireland, Roscrea, County of Tipperary.
Loudon, Mr. Surgeon.
M'Donnell, Mr. Surgeon, Acton-street, Gray's Inn.
Muriel, Mr. Surgeon, Cambridge.
Perfect and Podmore, Messrs. Town Malling.
Ray, Mr. George, Member of the Royal College of Surgeons, Milton, Kent.
Reid, Mr. Thomas, Surgeon and Accoucheur, Castle Street, Falcon-square.
Ring, Mr. Surgeon, Charlotte-street.
Shearman, Mr. Edward James, Surgeon, Rotherham.
Simmonds, Mr. B. Surgeon, Sherborne, Dorset.
Simpson, J. Esq. Ecclificham, N.B.
Spencer, Mr. Great Easton, Leicestershire.
Swinden, Mr. John, Surgeon, Morley, near Leeds.
Thatcher, John, M.D. Lecturer on Midwifery and the Diseases of Women and Children, Edinburgh.
Thomas, Mr. Surgeon, Carnarvon.
White, Mr. W. Member of the Royal College of Surgeons, and Surgeon to the City Infirmary and Dispensary of Bath.
Whicher, Mr. Medical Student, St. Thomas's and Guy's Hospitals.

---

CORRIGENDA IN THIS NUMBER.

Page 775, for *typographical,* read topographical.

Page 802, for *hydrodate,* read hydriodate.

ERRATA IN THE PHRENOLOGICAL ARTICLE.

Page 864, fourth line from bottom, for *compassion* read *comparison.*
Page 874, fourth line of note, for *shall* read *should.*
Page 881, nineteenth line from top, for *truth* read *resurrection.*

### INDEX.

Abdominal pressure........... 569
Abdominal tumour ........... 220
Abortion, Mr. Ward on ...... 699
Accretion of heart and pericardium ..................... 548
Acetate of morphia, preparation of ..................... 208
Acupuncture............. 956, 958
Alcock, Mr. on medical education ..................... 521
Allan, Mr. on acute mania.... 939
Amenorrhœa, Dr. Lavagna on . 700
Amputation, remarks on ..... 948
Amputation at the hip-joint .. 947
Amputation, tourniquets in .. 953
Anatomico - pathological researches................. 487
Anderson, Dr. on yellow fever 1009
Andral on the intestinal canal . 673
Aneurism, Mr. Todd on ...... 77
Aneurism mistaken.......... 919
Antimoniated ointment in agues 701
Aorta, aneurism of, Mr. Hunter 767
Apothecaries' transactions.... 519
Arsenic, poison of............ 221
Arson, Dr. Paris on.......... 601
Ascarides, remedy for........ 206
Ascension, fever at .......... 916
Asylum, Glasgow Lunatic .... 775
Axillary artery, rupture of.... 951
Baillie, Dr. estimate of his character.................... 734
Bann, contagious fever in .... 916
Barclay, Dr. answers to ...... 868
Bedlam, sketches in ......... 511
Bell, Mr. on cancer mammæ.. 195
Bile, Mr. Brodie on the ...... 186
Biliary calculi, Mr. Brayne on . 791
Biography of Dr. Baillie...... 734
Blood-letting in hydrothorax . 942
Blue disease, remarks on ...... 924
Bowman, Mr. on mercurial vapour....................... 1010
Brain, destruction of, in a fœtus 800
Brain, painful affection of .... 463
Brayne, Mr. on biliary calculi . 791
Briggs, Mr. James, on scirrhus and cancer ............... 172
Brodie, Mr. on the bile ...... 186
Brodie, Mr. on extraction of calculi..................... 804
Bronchocele, Mr. Austen on .. 193
Bronchocele, iodine in ....... 801
Bronchotomy ............... 213
Bronchotomy, Dr. Burgess on . 339
Brucine in paralysis ......... 944
Bull, Dr. on cancer of the lip.. 464
Burgess, Dr. on bronchotomy . 339
Burnett, Dr. William, on mercurial vapour ............. 1010
Byron, Dr. on coagulum of blood in the bladder.............. 316
Cæcum, ulceration of.... 470, 739
Cæcum, scirrhous, mistaken .. 919
Cæsarean operation.......... 964
Calculi, on extraction of, Sir A. Cooper ................... 803
Cancer, Professor Scarpa on .. 172
Cancer, Mr. Bell on.......... 195
Cancer, cured by oxymurias hydrarg..................... 222
Cancer, chimney-sweepers.... 799
Cancer of the penis .......... 837
Carbuncle, Mr, Swallow...... 934
Carmichael, Mr. on strangulated hernia ............... 340
Carmichael, Mr. on phrenology 868
Carotid artery, wound of...... 334
Carter, Dr. on epilepsy ....... 700
Carter, Dr. on diabetes ...... 935
Cas Rares, or remarkable cases 295
Castaing, Dr. his case considered .................... 968
Catarrhus vesicæ ............ 749
Cathartics, Dr. Chapman on.. 72
Chapman, Dr. on therapeutics 61, 551
Cheyne, Dr. on dysentery .... 136
Cholera epidemica .......... 927
Circulation, physiology of .... 38
Circulation of the blood ...... 966
Clarke, Mr. on female diseases 635
Coagulum of blood in the bladder ...................... 316
Coal tar, how far a cause of foul air ....................... 996
College of Physicians ........ 386
College of Surgeons.......... 390
Combe, Mr. George, on phrenology.................. 848, 855
Colles, Dr. on dissection wounds 95
Colon, stricture of........... 451
Colon, disease of, simulating aneurism of the heart...... 468
Constipation, Dr. Maxwell on . 937
Constipation, Dr. Chisholm on 941
Contagion of yellow fever, Dr. Musgrave on.............. 979

Cooke, Dr. on epilepsy ...... 119
Cooper, Sir Astley, on dilatation of urethra ............... 193
Cooper, Sir Astley, on fractures 707
Cooper, Sir A. on extraction of calculi ................. 803
Countenance, signs of in disease 813
Crampton, Dr. Philip, on leeching internal surfaces ...... 313
Crichton, Sir Alex. on phthisis 102
Crichton, Dr. on yellow fever.. 1002
Cubebs in gonorrhœa ........ 216
Cuming, Dr. on disease of the heart ..................... 331
Cusack, Dr. ligature of the carotid ..................... 334
Cutaneous diseases, Mr. Wilkinson on ................ 132
Dasher, transport, fever in.... 994
Davy, Dr. on pneumo-thorax.. 922
Death, sudden and mysterious . 920
Delirium tremens ........... 918
Diabetes mellitus ......... 200, 345
Diabetes ................... 935
Diagnosis, Dr. Hall on......... 811
Diaphragmatic pleuritis...... 912
Diaphoretics, observations on . 76
Dislocations, Sir A. Cooper on 225
Dissections, wounds in ...... 95
Distortions of the spine, Mr. Shaw on.................. 882
Diuretics, Dr. Chapman on.. 74
Douglas, Dr. on puerperal fever 83
Douglas, Dr. on extraction of the placenta .............. 961
Dracunculus, Dr. Scott........ 182
Dublin, on the diseases of.... 88
Dublin Hosp. Reports, Vol. III. 313
Dupuytren, M. on prolapsus ani 194
Dyett, Dr. on yellow fever ... 1008
Dysentery, Cheyne and O'Brien on..................... 136
Earle, Mr. H. observations in surgery .................. 707
Earle, Mr. on ununited fractures 192
Ectropion, Mr. Guthrie on.... 437
Education, medical, Mr. Alcock on ..................... 521
Ellis, Mr. biography of Dr. Gordon ................... 29
Emetics, Dr. Chapman on .... 69
Enteritis, Mr. Wade on ...... 193
Enteritis, M. Tacheron on ... 743
Entropium, Mr Guthrie on 429, 658
Epilepsy, Dr. Cooke on ...... 119
Epilepsy, Baron Larrey on.... 465
Epilepsy, Dr. Carter on ...... 700
Esophagus, stricture of ...... 237
Esquirol on suicide .......... 1
Evans, Mr. on mal-practices in medicine ................ 768
Extirpation of the thyroid gland 702
Extravasation within the cranium ................. 952
Eye, lectures on, Mr. Guthrie . 427
Falret, Dr. on suicide ........ 1
Females, on the diseases of ... 635
Femoral aneurism, spontaneous cure of .................. 955
Fever, bilious remittent ...... 211
Fever, yellow, Dr. O'Halloran 282
Fever, yellow, in the Bann and at Ascension .............. 916
Fleurens, M. on the nervous system .................. 703
Fœtus, destruction of brain in . 800
Fœtus, venereal disease in.... 839
Food, adulteration of ........ 599
Fractures of the cranium (Mr. Baker) .................. 953
Fractures, diagnosis of ...... 954
Fractures ununited, Mr. Earle 192
Glasgow Lunatic Asylum ..... 775
Glottis, on œdema of the .... 452
Good, Dr. study of medicine .. 155
Gordon, Dr. biography of .... 29
Gregory, Dr. George, on post-vacine disease ............ 806
Gregory's, Dr. George, case of encysted tumour ........... 915
Guthrie, Mr. operative surgery of the eye ............ 427, 658
Haden, Mr. on the poison of food ..................... 765
Hæmatemesis ............... 928
Hæmoptysis ................ 157
Hæmorrhage, internal........ 930
Hall, Dr. on Semeiology...... 811
Hammond, Mr. on destroyed brain in a fœtus .......... 800
Hartle, inspector, his reports on yellow fever .............. 993
Harvey, curious preparations of 234
Hawkins, Mr. on ulcerations of the larynx ................ 471
Heart, disease of, Dr. Cuming 331
Heart, enlarged ............ 706
Heart, chronic inflammation of 548
Herley, Mr. on a paralytic affection ................ 318
Hernia, Mr. Lizars on the treatment of .................. 232
Hey, Mr. his life, by Mr. Pearson 827
Hip-joint, dislocations of .... 342
Homicide ................. 607
Homicide, trial for .......... 969
Hooping-cough, new theory of 449
Hospital reports (Dub.) Vol. III. 77
Howship, Mr. obstructed œsophagus ................. 317
Howship, Mr. on the urinary organs .................. 339

Human combustion.......... 604
Hume, Dr. on tonic treatment of phthisis................ 189
Hydatid, Mr. Pretty's case.... 930
Hydrophobia, Marochetti on .. 917
Hydrophobia, supposed case of 942
Hydrothorax, M. Tacheron on 757
Hypochondriasis ............ 162
Infanticide, trial for........... 972
Infants, on the vitality of..... 738
Inflammation, Dr. Lucas on.. 38
Inflammation, products of.... 223
Inflammation of the diaphragmatic pleura............... 912
Inflammations, cutaneous .... 489
Injecting machine for the stomach .................... 742
Insanity, traits of, in Bedlam.. 511
Internal surfaces, leeches to .. 313
Intestine, portion of, expelled . 932
Irritation, Mr. Earle on ...... 793
Jaundice, Dr. Henry Marsh on 319
Jaw, on injury of, Mr. Powell . 766
Johnson, Dr. on disease of the heart ..................... 548
Joints, on fractures, &c. of. .. 707
Journal of phrenology......... 847
Journals, medical, increase of . 911
Jurisprudence, medical.....383, 596
Kerrison, Dr. on dilatation of the urethra ............... 802
Knife-eater, account of ...... 218
Lachrymal gland, diseases of.. 344
Lancet, (Medical Journal) examined ..................... 973
Larrey, Baron, on epilepsy.... 465
Laryngeal disease, Mr. Shaw on 188
Larynx, remarkable disease of. 452
Larynx, syphilitic ulcers of.... 471
Lecturers, their privileges discussed ..................... 973
Leeches, Dr. Price on........ 697
Leeches to internal surfaces, 313, 940
Legal medicine.............. 968
Lip, cancer of, Dr. Bull ...... 464
Lips, on diseases of .. ....... 794
Lithontriptics, Dr. Chapman on 75
Lizars, Mr. on the treatment of hernia ................... 232
Local irritation, Mr. Earle on, 793
Lucas, Dr. on inflammation and fever ..................... 38
Lunacy, destructive propensities in.................... 781
Lunatic Asylum of Glasgow ... 775
Magendie, Dr. on the spinal marrow ................... 702
Mammæ, on enlargement of.. 838
Marsh, Dr. on jaundice ...... 319
Marsh, Dr. on diabetes ...... 345
Materia medica, Dr. Chapman on ...................... 61
Materialism and immaterialism 159
Medical practitioners, actions against ................. 391
Medical jurisprudence ....426, 596
Medico-Chirurg. Transactions . 790
Meningine, Dr. Scoulton on .. 698
Mercurial vapour, Dr. Burnett on .......................1010
Mercury in acute diseases .... 222
Mercury, Mr. Swan on the action of ................... 277
Merriman, Dr. on the vitality of infants ............... 738
Muscle, degeneration of...... 945
Musgrave, Dr. on yellow fever 979
Nerve, wounds of............ 550
Nerves, on painful affections of 766
Nervous system, physiology of, 966
Nervous system. M. Fleurens on 703
Neuralgia .................. 167
Nuisances.................. 419
O'Beirne, Dr. on tetanus...... 335
O'Brien, Dr. on dysentery .... 136
Obstetric delinquency........ 961
Œdema of the glottis........ 452
Œsophagotomy ............. 947
Œsophagus, case of obstructed 317
O'Halloran, Dr. on yellow fever 282
Opium injected into the veins . 935
Opium in acute mania........ 939
Oxalic acid, experiments on .. 201
Paralysis, Dr. Prichard on .... 926
Paralytic affection, curious case of ...................... 318
Paris, Dr. on medical jurisprudence ..............383, 596
Parry, Dr. on the pulse ...... 33
Pathology, Dr. Pring on...... 241
Pathology of the intestinal canal 673
Pearson, Mr. his life of Mr. Hey 827
Penis, cancer of.............. 837
Penitentiary at Milbank...... 235
Percival, Mr. on veterinary medicine.................... 583
Perineum, perforation of...... 318
Periscope, on the importance of 911
Peritonitis, supposed from poison ..................... 970
Petechiæ hæmorrhagicæ...... 206
Pett, Dr. his death ........... 98
Pharmaceutical formulæ ..... 938
Phrenology, curious case of .. 969
Phrenological Society's Transactions ................... 848
Phrenological Journal........ 847
Phthisis, Sir A. Crichton on .. 102
Phthisis, tonic treatment of.. 189
Phthisis pulmonalis, Mr. Gaitskell on .................. 929

Physiological pathology ...... 934
Physiology of the circulation.. 38
Placenta, extraction of ...... 961
Plethora, Dr. Good on........ 156
Pleuritis, M. Tacheron on .... 754
Pneumonia, Dr. Forbes on .... 928
Poison of food, Mr. Haden .... 765
Poisoning from nux vomica... 972
Poole, Dr. on phrenology .... 851
Popular medicine, Dr. Thomas 238
Powell, Mr. on injury of the jaw 766
Pretty, Mr. on petechiæ hæmorrhagicæ ................. 206
Prichard, Dr. on paralysis .... 926
Price, Dr. on leeches ...... .. 697
Pring, Dr. on general pathology 241
Prolapsus ani, treatment of... 194
Prussic acid ................ 940
Puerperal fever, Dr. Douglas on 83
Puerperal convulsions......... 963
Purpura hæmorrhagica ....... 941
Putrefaction, corrector of .... 939
Pyramus frigate, fever in...... 995
Rape ..................... 606
Retention of urine .......... 959
Rheumatic metastasis......... 215
Rheumatism, on the treatment of ........................ 933
Roots, Dr. on bronchocele .... 801
Salivation, mode of arresting.. 941
Sanguineous tumour, Mr. Briggs 180
Scarpa on scirrhus and cancer 172
Scirrhus, Professor Scarpa on.. 172
Scott, Dr. on Dracunculus..... 182
Scott, Dr. on dislocations of the hip joint ................. 342
Scott, Mr. on phrenology ..... 854
Semeiology, Dr. Hall on....... 811
Shaw, Mr. on laryngeal disease 188
Shaw, Mr. on distortions of the spine .................... 882
Shillito, Mr. on ruptured uterus 762
Simulated diseases .......... 596
Slow poisons............... 627
Speer, Dr. on the diseases of Dublin ................... 88
Spinal marrow, destruction of, 701
Sprague's, Mr. formulæ ...... 938
St. Kitts, yellow fever there .. 993
Strangulated hernia, curious case of ................ 340
Strychnine and brucine ....... 944
Subclavian artery, ligature of.. 954
Subclavian aneurism ........ 958
Suicide, Esquirol and Falret on 1
Suicide, Dr. Paris on ........ 625
Surgical operations, Mr. Averell on ................ 945
Swan, Mr. on the action of mercury .................... 277
Symphysis pubis, division of .. 965
Symptomatology, Dr. Hall on.. 811
Tacheron's pathological researches ................487, 743
Taliacotian operation ........ 960
Tar vapour, Sir A. Crichton on 102
Tetanus, Mr. Hutchinson on.. 200
Tetanus, Dr. O'Beirne on .... 335
Tetanus, treatment of......... 933
Therapeutics, Dr. Chapman on 61
Therapeutics................. 935
Therapeutics, elements of .... 551
Thigh-bone, fractures of...... 707
Thoracic percussion, on...... 569
Thyroid gland, extirpation of.. 702
Tic douloureux, iron in ...... 700
Todd, Mr. on aneurism ...... 77
Todd, Mr. on diseases of lachrymal gland ............... 344
Tourniquets in amputation ... 953
Tracheotomy, curious case of.. 955
Transactions of the Apothecaries...................... 519
Transactions of the Med.-Chir- Society .................. 790
Transactions of the Phrenological Society................ 847
Triumph (74) mercurial vapour in ......................1010
Tumour, encysted, curious case 915
Tumour, immense, removed, (Mr. Liston) .............. 957
Ulceration of the cæcum ..... 739
Urethra (female) dilatation of, 193
Urethra of the male, Dr. Kerrison ..................... 802
Urinary organs, Mr. Howship on ....................... 369
Urinary secretion, physiology of 965
Urine, curious case of suppression ...................... 372
Urine, suppression of ........ 959
Urine, retention of .......... 959
Uterus, cancer of, extirpation 463
Uterus, rupture of, Mr. Shillito 762
Uvula, elongation of ........ 956
Vaccination, Dr. Gregory on.. 606
Vaccine and measles at once.. 205
Varicose veins .............. 954
Variola and varicella ........ 214
Venereal disease in the fœtus .. 839
Veterinary art, lectures on.... 583
Vincent, Mr. operation on the neck ..................... 207
Wardrop, Mr. on wounded nerves .................... 450
Webster, Dr. on hooping-cough 449
Wilkinson, Mr. on Cutaneous diseases .................. 132
Wounds, poisoned .......... 698
Yellow fever, Dr. Musgrave on 979
Yellow fever, Dr. Hartle on .. 993

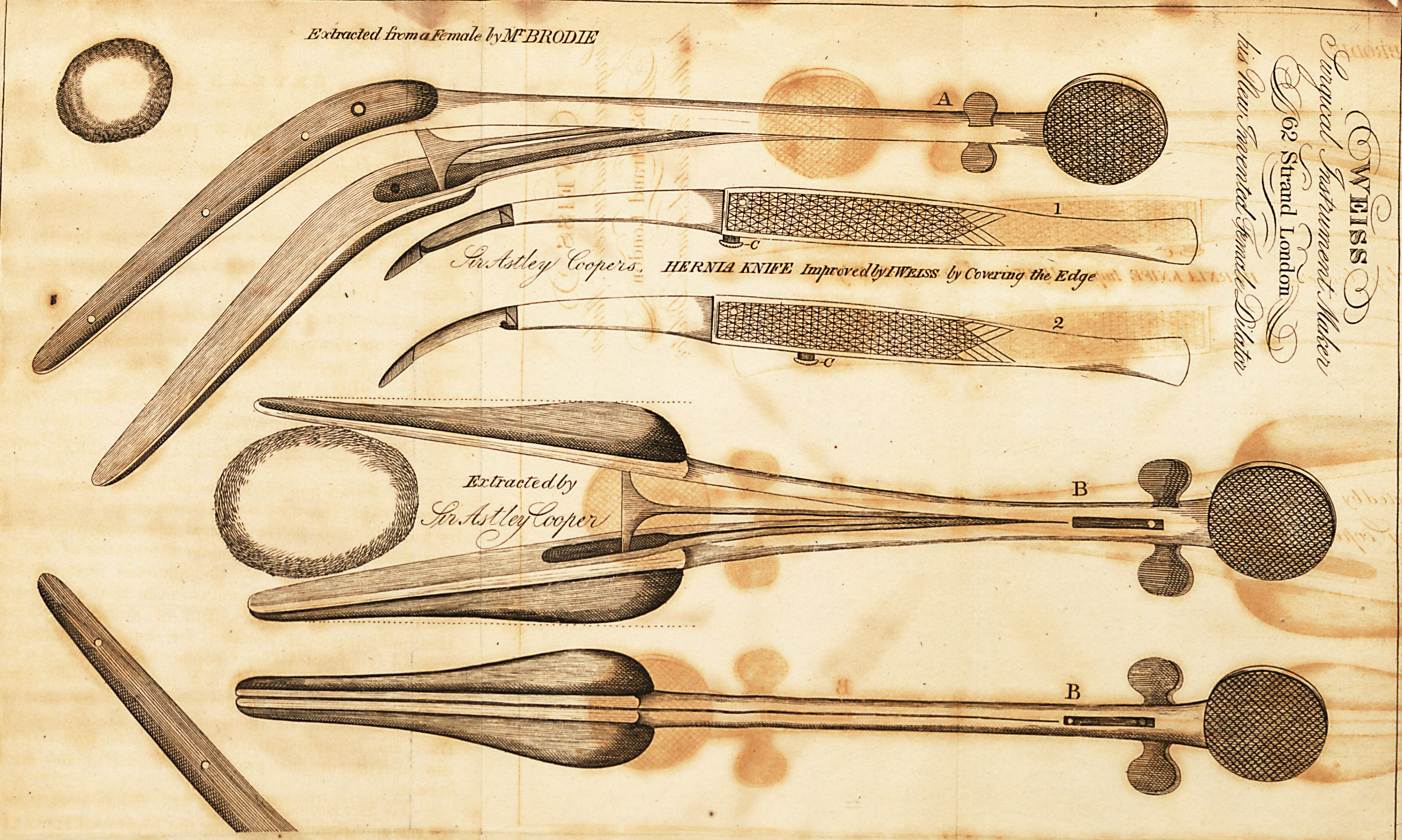

Extracted from a Female by Mr. BRODIE

A

1

C

Sir Astley Coopers, HERNIA KNIFE Improved by I. WEISS by Covering the Edge

2

C

Extracted by

Sir Astley Cooper

B

B

WEISS

Surgical Instrument Maker

62 Strand London his New Invented Female Dilator

### EXPLANATION

OF

### J. WEISS'S

### *Female Dilators and Improved Hernia Knife.*

The Female Dilator, by turning the handle to the right, will be found to open to any extent you may think proper. The Instrument A has a curve in it, which by some gentlemen is thought an improvement, as it does not take up so much room. The Hernia Knife, letter C, has a small button, which, when you wish to cover the cutting part of the Instrument, you must press with your finger and push it forward.

---

Mr. Weiss has the honor of laying before the profession, engravings of some Instruments lately invented by himself, and he is happy to say, approved of by several of the most distinguished Surgeons of the metropolis, as will be seen by the annexed letters. He has also to refer to the 12th volume of the Medico-Chirurgical Transactions, or to page 193 of the 13th No. of the Medico-Chirurgical Review, for some interesting cases published by Sir Astley Cooper, respecting the Female Dilator. Mr. Weiss feels it a justice to add, that the Female Dilator was suggested to him by that distinguished ornament of the profession, Sir Astley Cooper, Bart. and it is most gratifying to his feelings to find that the Instrument has proved so useful in the hands of others.

To Mr. Bransby Cooper Mr. Weiss is also indebted for the suggestion respecting the Improved Hernia Knife, Nos. 1 and 2 in the Plate; which gentleman very frequently mentioned to him that it would be a very great improvement if the cutting part could be covered when introduced: which he has effected to Mr. B. Cooper's great satisfaction.

22, *Lincoln's Inn Fields*,

*May* 16*th*, 1823.

Sir,

I have great pleasure in offering my testimony to the utility of your Instrument for dilating the female Urethra, and I shall be happy if the following case should contribute to recommend it to the notice of Surgeons.

Mary Wallace, thirty-one years of age, of a delicate frame and irritable constitution, was admitted into St. Thomas's Hospital on the 24th of last October, with well marked symptoms of stone in the bladder. The presence of the calculus was ascertained by sounding; but the operation was deferred, in consequence of her being out of health, until the 22d of November. On that day, I introduced the dilator, and separated the blades until the patient complained of pain; and then waiting till the uneasiness had subsided, I again increased the expansion of the instrument, by means of the screw, till pain was reproduced. In the course of a few minutes the painful sense of stretching and distension was relieved, and I proceeded with the dilatation. In this manner, regulated by the feelings of my patient, I continued gradually and cautiously to enlarge the meatus during two hours and a half. I was then enabled to introduce with ease my fore finger into the bladder; and finding that the calculus was in a favorable position, I introduced a pair of straight forceps, without waiting for further dilatation, and readily seized the stone in its long axis. I proceeded immediately to the extraction; but from the size of the calculus, I was unable to bring it readily through the meatus, and during this attempt she complained of much greater pain than at any time during the dilatation. As I held the stone in the most favorable position, I was however unwilling to quit my hold, and with moderate force, (at least with no more than is ordinarily required, and in no longer time than is frequently necessary, in extracting a large sized stone in the operation of lithotomy,) I succeeded in extricating the calculus. It proved to be of considerable size, as the subjoined accurate admeasurement will fully shew.

She seemed much exhausted by the operation; and this was followed during the first few days by considerable constitutional irritation, and by symptoms indicating abdominal inflammation. Under the prompt and active measures however, which were taken, she was gradually restored to health. It is particularly worthy of notice, that she passed her urine freely during the whole time; and that she was able, in the course of twelve hours after the operation, to hold her urine more or less perfectly, and to discharge it voluntarily; and that the dilatation of the meatus left ultimately no weakness nor loss of power in retaining her urine.

The above case is, I think, a gratifying proof of the facility with which even a large calculus may be extracted by the aid of the Dilator. The operation might have been effected with less inconvenience had the dilatation been carried on somewhat longer; and I do not doubt that the enlargement of the meatus might be accomplished in much less time, without any difference in the result. The subsequent inflammatory affection and accompanying constitutional irritation I should be disposed to attribute much more to a peculiarity in the constitution of the female, than to any circumstances necessarily connected with the operation. The result of this case, as that of others, evinces, in the circumstance that the patient was relieved without producing incontinence of urine, a decided superiority to the use of the knife, as it regards this essential point.

I am, Sir,
Your obedient Servant,
JOSEPH HENRY GREEN.

Mr. WEISS,
Surgeon's Instrument Maker,
STRAND.

---

*Pall Mall*, 17*th April* 1823.

SIR,

I have the satisfaction to inform you, I have used the Instrument you have invented for dilating the *female* Urethra, and I have found it answer extremely well. I dilated the Urethra with great ease to allow of the introduction of a pair of Forceps, with which I extracted a stone larger than a French bean of the mulberry kind, which had been in the bladder four years, and had given the woman great pain; the bladder for many months was so irritable, she was obliged to use a Catheter to prevent the acute pain she always felt from the efforts made to expel urine.

I consider your Instrument of great utility, and I hope it will be generally used; and that the female part of the creation will in future (by an early use of it) avoid the usual operation which was almost always followed by an involuntary discharge of urine during the remainder of their lives.

I am, Sir,
Your obedient Servant,
JOHN PHILLIPS.

To Mr. WEISS.

---

16, *Saville Row*, *May* 21, 1823.

SIR,

I extracted the calculus which I shewed you lately, from a female patient in St. George's Hospital, by the process of dilating the urethra. I employed for this purpose, the Instrument which you invented, and which has been used and recommended by Sir Astley Cooper. The dilatation was effected very readily, but gradually, in the space of twenty-four hours. The patient suffered little inconvenience afterwards, except from incontinence of urine, which however, ceased to trouble her in less than a fortnight from the time of the operation.

I am, Sir,

Your obedient Servant,

B. C. BRODIE.

Mr. WEISS,
62, STRAND.

---

J. BURSLEM, Printer, Blackfriars Road.

